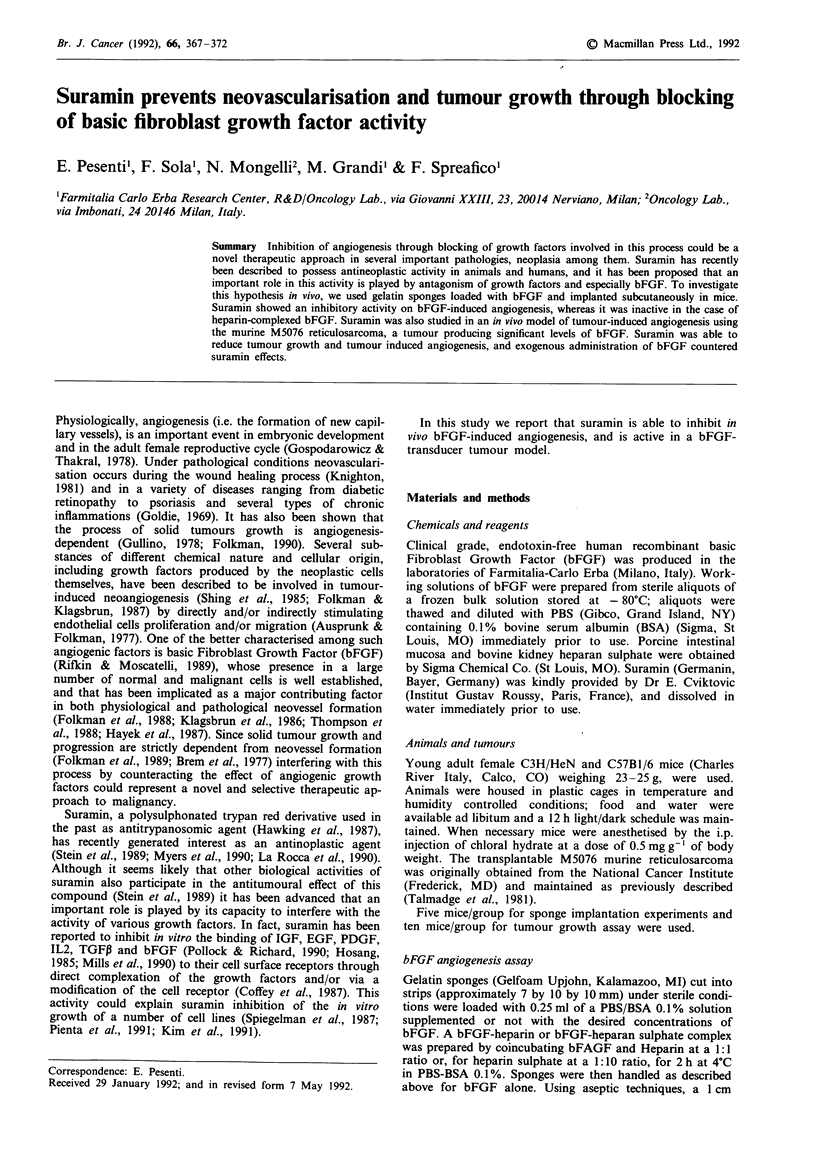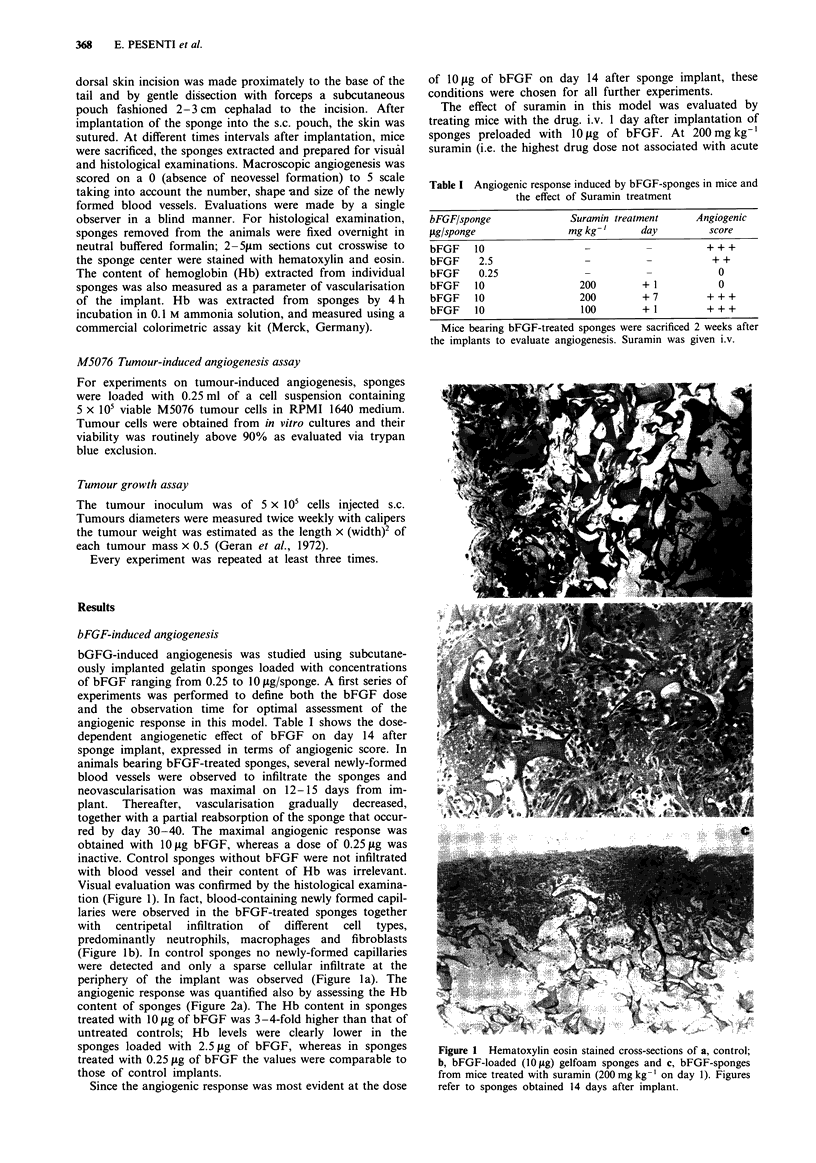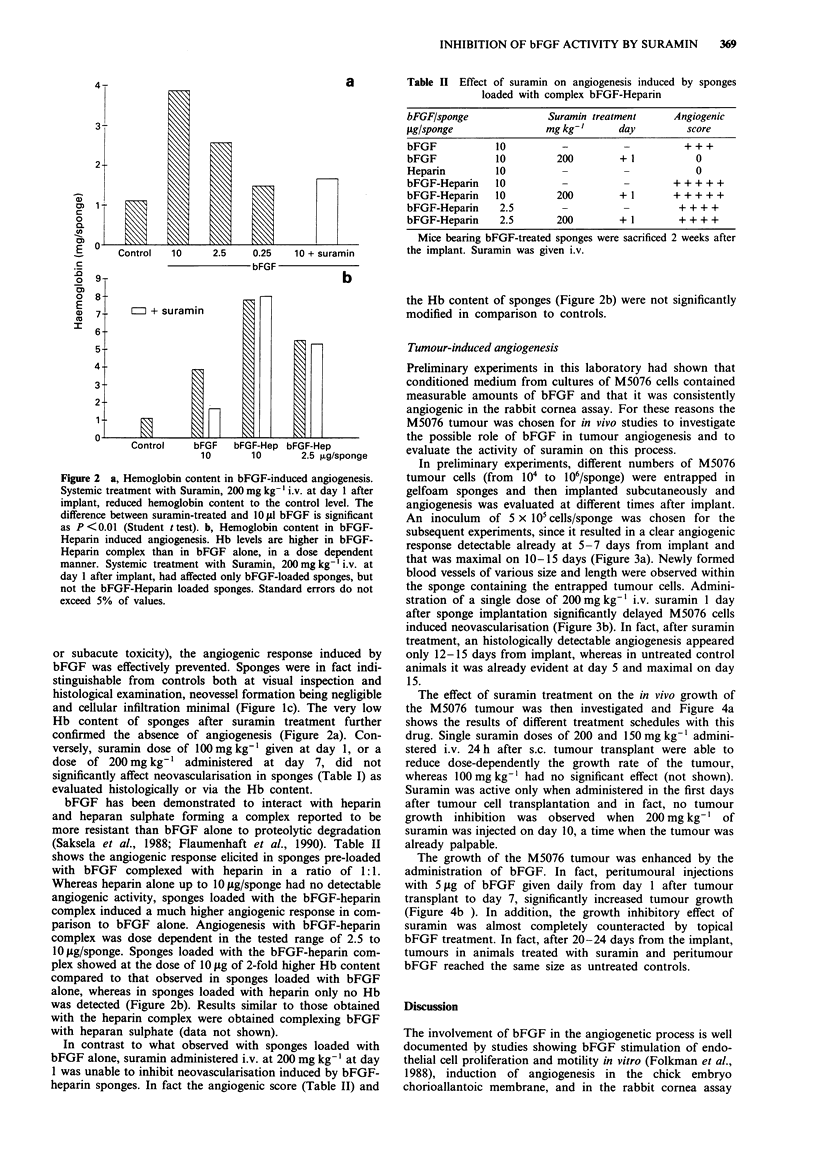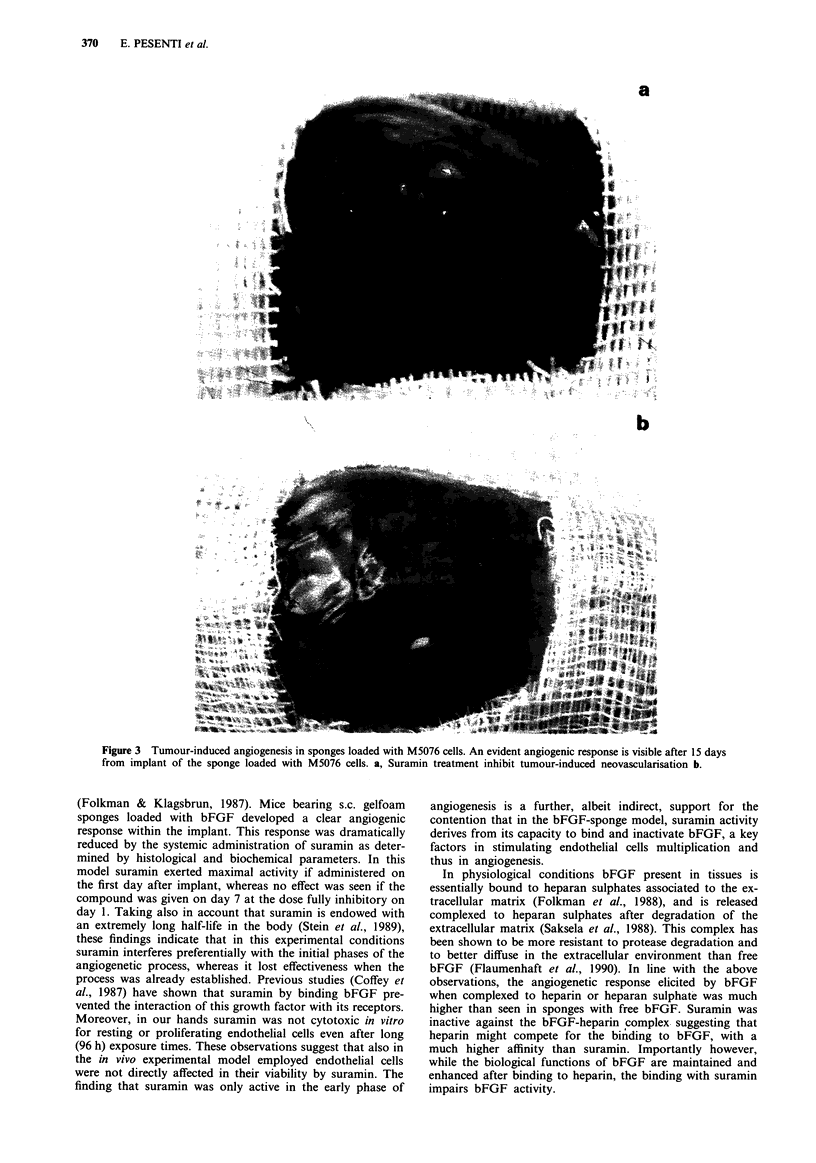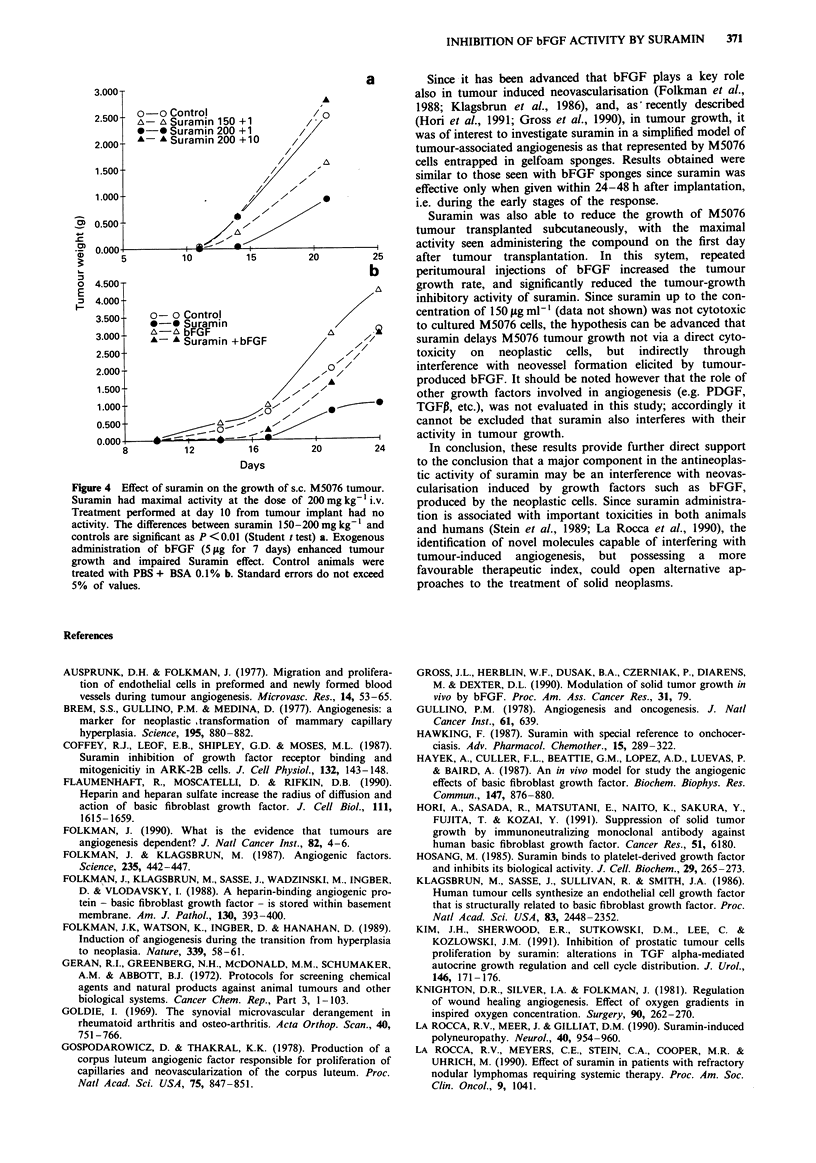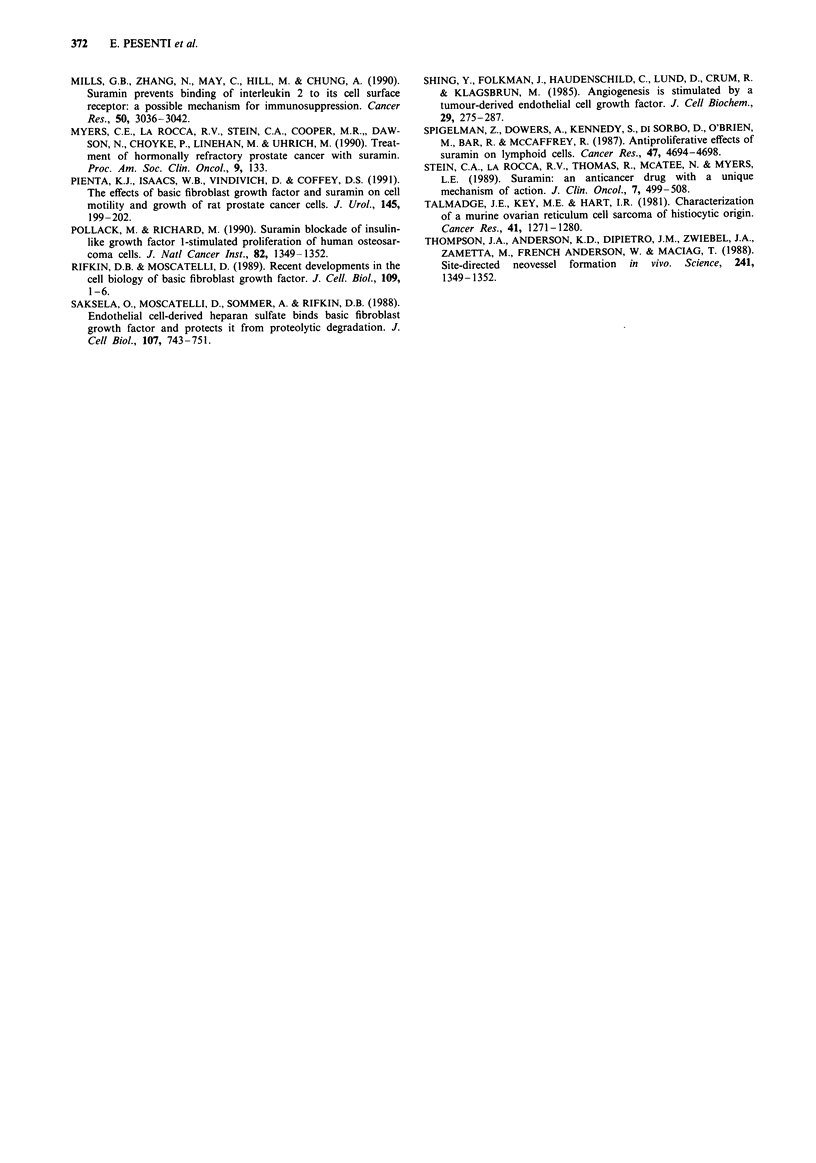# Suramin prevents neovascularisation and tumour growth through blocking of basic fibroblast growth factor activity.

**DOI:** 10.1038/bjc.1992.272

**Published:** 1992-08

**Authors:** E. Pesenti, F. Sola, N. Mongelli, M. Grandi, F. Spreafico

**Affiliations:** Farmitalia Carlo Erba Research Center, R&D/Oncology Lab., Milan, Italy.

## Abstract

**Images:**


*Br. J. Cancer* (1992), **66**, 367–372 © Macmillan Press Ltd., 1992

### Suramin prevents neovascularisation and tumour growth through blocking of basic fibroblast growth factor activity

E. Pesenti[1], F. Sola[1], N. Mongelli[2], M. Grandi[1] & F. Spreafico[1]

[1]*Farmitalia Carlo Erba Research Center, R&D/Oncology Lab., via Giovanni XXIII, 23, 20014 Nerviano, Milan;* [2]*Oncology Lab., via Imbonati, 24 20146 Milan, Italy.*

**Summary** Inhibition of angiogenesis through blocking of growth factors involved in this process could be a novel therapeutic approach in several important pathologies, neoplasia among them. Suramin has recently been described to possess antineoplastic activity in animals and humans, and it has been proposed that an important role in this activity is played by antagonism of growth factors and especially bFGF. To investigate this hypothesis *in vivo*, we used gelatin sponges loaded with bFGF and implanted subcutaneously in mice. Suramin showed an inhibitory activity on bFGF-induced angiogenesis, whereas it was inactive in the case of heparin-complexed bFGF. Suramin was also studied in an *in vivo* model of tumour-induced angiogenesis using the murine M5076 reticulosarcoma, a tumour producing significant levels of bFGF. Suramin was able to reduce tumour growth and tumour induced angiogenesis, and exogenous administration of bFGF countered suramin effects.

Physiologically, angiogenesis (i.e. the formation of new capillary vessels), is an important event in embryonic development and in the adult female reproductive cycle (Gospodarowicz & Thakral, 1978). Under pathological conditions neovascularisation occurs during the wound healing process (Knighton, 1981) and in a variety of diseases ranging from diabetic retinopathy to psoriasis and several types of chronic inflammations (Goldie, 1969). It has also been shown that the process of solid tumours growth is angiogenesis-dependent (Gullino, 1978; Folkman, 1990). Several substances of different chemical nature and cellular origin, including growth factors produced by the neoplastic cells themselves, have been described to be involved in tumour-induced neoangiogenesis (Shing *et al.*, 1985; Folkman & Klagsbrun, 1987) by directly and/or indirectly stimulating endothelial cells proliferation and/or migration (Ausprunk & Folkman, 1977). One of the better characterised among such angiogenic factors is basic Fibroblast Growth Factor (bFGF) (Rifkin & Moscatelli, 1989), whose presence in a large number of normal and malignant cells is well established, and that has been implicated as a major contributing factor in both physiological and pathological neovessel formation (Folkman *et al.*, 1988; Klagsbrun *et al.*, 1986; Thompson *et al.*, 1988; Hayek *et al.*, 1987). Since solid tumour growth and progression are strictly dependent from neovessel formation (Folkman *et al.*, 1989; Brem *et al.*, 1977) interfering with this process by counteracting the effect of angiogenic growth factors could represent a novel and selective therapeutic approach to malignancy.

Suramin, a polysulphonated trypan red derivative used in the past as antitrypanosomic agent (Hawking *et al.*, 1987), has recently generated interest as an antinoplastic agent (Stein *et al.*, 1989; Myers *et al.*, 1990; La Rocca *et al.*, 1990). Although it seems likely that other biological activities of suramin also participate in the antitumoural effect of this compound (Stein *et al.*, 1989) it has been advanced that an important role is played by its capacity to interfere with the activity of various growth factors. In fact, suramin has been reported to inhibit *in vitro* the binding of IGF, EGF, PDGF, IL2, TGF$\beta$ and bFGF (Pollock & Richard, 1990; Hosang, 1985; Mills *et al.*, 1990) to their cell surface receptors through direct complexation of the growth factors and/or via a modification of the cell receptor (Coffey *et al.*, 1987). This activity could explain suramin inhibition of the *in vitro* growth of a number of cell lines (Spiegelman *et al.*, 1987; Pienta *et al.*, 1991; Kim *et al.*, 1991).

Correspondence: E. Pesenti.
Received 29 January 1992; and in revised form 7 May 1992.

In this study we report that suramin is able to inhibit *in vivo* bFGF-induced angiogenesis, and is active in a bFGF-transducer tumour model.

### Materials and methods

### *Chemicals and reagents*

Clinical grade, endotoxin-free human recombinant basic Fibroblast Growth Factor (bFGF) was produced in the laboratories of Farmitalia-Carlo Erba (Milano, Italy). Working solutions of bFGF were prepared from sterile aliquots of a frozen bulk solution stored at − 80°C; aliquots were thawed and diluted with PBS (Gibco, Grand Island, NY) containing 0.1% bovine serum albumin (BSA) (Sigma, St Louis, MO) immediately prior to use. Porcine intestinal mucosa and bovine kidney heparan sulphate were obtained by Sigma Chemical Co. (St Louis, MO). Suramin (Germanin, Bayer, Germany) was kindly provided by Dr E. Cviktovic (Institut Gustav Roussy, Paris, France), and dissolved in water immediately prior to use.

### *Animals and tumours*

Young adult female C3H/HeN and C57B1/6 mice (Charles River Italy, Calco, CO) weighing 23–25 g, were used. Animals were housed in plastic cages in temperature and humidity controlled conditions; food and water were available ad libitum and a 12 h light/dark schedule was maintained. When necessary mice were anesthetised by the i.p. injection of chloral hydrate at a dose of 0.5 mg $g^{-1}$ of body weight. The transplantable M5076 murine reticulosarcoma was originally obtained from the National Cancer Institute (Frederick, MD) and maintained as previously described (Talmadge *et al.*, 1981).

Five mice/group for sponge implantation experiments and ten mice/group for tumour growth assay were used.

### *bFGF angiogenesis assay*

Gelatin sponges (Gelfoam Upjohn, Kalamazoo, MI) cut into strips (approximately 7 by 10 by 10 mm) under sterile conditions were loaded with 0.25 ml of a PBS/BSA 0.1% solution supplemented or not with the desired concentrations of bFGF. A bFGF-heparin or bFGF-heparan sulphate complex was prepared by coincubating bFAGF and Heparin at a 1:1 ratio or, for heparin sulphate at a 1:10 ratio, for 2 h at 4°C in PBS-BSA 0.1%. Sponges were then handled as described above for bFGF alone. Using aseptic techniques, a 1 cm dorsal skin incision was made proximately to the base of the tail and by gentle dissection with forceps a subcutaneous pouch fashioned 2–3 cm cephalad to the incision. After implantation of the sponge into the s.c. pouch, the skin was sutured. At different times intervals after implantation, mice were sacrificed, the sponges extracted and prepared for visual and histological examinations. Macroscopic angiogenesis was scored on a 0 (absence of neovessel formation) to 5 scale taking into account the number, shape and size of the newly formed blood vessels. Evaluations were made by a single observer in a blind manner. For histological examination, sponges removed from the animals were fixed overnight in neutral buffered formalin; 2–5µm sections cut crosswise to the sponge center were stained with hematoxylin and eosin. The content of hemoglobin (Hb) extracted from individual sponges was also measured as a parameter of vascularisation of the implant. Hb was extracted from sponges by 4 h incubation in 0.1 M ammonia solution, and measured using a commercial colorimetric assay kit (Merck, Germany).

*M5076 Tumour-induced angiogenesis assay*

For experiments on tumour-induced angiogenesis, sponges were loaded with 0.25 ml of a cell suspension containing $5 \times 10^5$ viable M5076 tumour cells in RPMI 1640 medium. Tumour cells were obtained from *in vitro* cultures and their viability was routinely above 90% as evaluated via trypan blue exclusion.

*Tumour growth assay*

The tumour inoculum was of $5 \times 10^5$ cells injected s.c. Tumours diameters were measured twice weekly with calipers the tumour weight was estimated as the length × $(\text{width})^2$ of each tumour mass × 0.5 (Geran *et al.*, 1972).

Every experiment was repeated at least three times.

### Results

*bFGF-induced angiogenesis*

bGFG-induced angiogenesis was studied using subcutaneously implanted gelatin sponges loaded with concentrations of bFGF ranging from 0.25 to 10 µg/sponge. A first series of experiments was performed to define both the bFGF dose and the observation time for optimal assessment of the angiogenic response in this model. Table I shows the dose-dependent angiogenetic effect of bFGF on day 14 after sponge implant, expressed in terms of angiogenic score. In animals bearing bFGF-treated sponges, several newly-formed blood vessels were observed to infiltrate the sponges and neovascularisation was maximal on 12–15 days from implant. Thereafter, vascularisation gradually decreased, together with a partial reabsorption of the sponge that occurred by day 30–40. The maximal angiogenic response was obtained with 10 µg bFGF, whereas a dose of 0.25 µg was inactive. Control sponges without bFGF were not infiltrated with blood vessel and their content of Hb was irrelevant. Visual evaluation was confirmed by the histological examination (Figure 1). In fact, blood-containing newly formed capillaries were observed in the bFGF-treated sponges together with centripetal infiltration of different cell types, predominantly neutrophils, macrophages and fibroblasts (Figure 1b). In control sponges no newly-formed capillaries were detected and only a sparse cellular infiltrate at the periphery of the implant was observed (Figure 1a). The angiogenic response was quantified also by assessing the Hb content of sponges (Figure 2a). The Hb content in sponges treated with 10 µg of bFGF was 3–4-fold higher than that of untreated controls; Hb levels were clearly lower in the sponges loaded with 2.5 µg of bFGF, whereas in sponges treated with 0.25 µg of bFGF the values were comparable to those of control implants.

Since the angiogenic response was most evident at the dose of 10 µg of bFGF on day 14 after sponge implant, these conditions were chosen for all further experiments.

The effect of suramin in this model was evaluated by treating mice with the drug. i.v. 1 day after implantation of sponges preloaded with 10 µg of bFGF. At 200 mg $kg^{-1}$ suramin (i.e. the highest drug dose not associated with acute

**Table I** Angiogenic response induced by bFGF-sponges in mice and the effect of Suramin treatment

| *bFGF/sponge µg/sponge* | *Suramin treatment mg kg⁻¹* | *day* | *Angiogenic score* |
|---|---|---|---|
| bFGF 10 | – | – | + + + |
| bFGF 2.5 | – | – | + + |
| bFGF 0.25 | – | – | 0 |
| bFGF 10 | 200 | + 1 | 0 |
| bFGF 10 | 200 | + 7 | + + + |
| bFGF 10 | 100 | + 1 | + + + |

Mice bearing bFGF-treated sponges were sacrificed 2 weeks after the implants to evaluate angiogenesis. Suramin was given i.v.

**Figure 1** Hematoxylin eosin stained cross-sections of **a**, control; **b**, bFGF-loaded (10 µg) gelfoam sponges and **c**, bFGF-sponges from mice treated with suramin (200 mg $kg^{-1}$ on day 1). Figures refer to sponges obtained 14 days after implant.

**Figure 2** a, Hemoglobin content in bFGF-induced angiogenesis. Systemic treatment with Suramin, 200 mg $kg^{-1}$ i.v. at day 1 after implant, reduced hemoglobin content to the control level. The difference between suramin-treated and 10 µl bFGF is significant as $P < 0.01$ (Student *t* test). b, Hemoglobin content in bFGF-Heparin induced angiogenesis. Hb levels are higher in bFGF-Heparin complex than in bFGF alone, in a dose dependent manner. Systemic treatment with Suramin, 200 mg $kg^{-1}$ i.v. at day 1 after implant, had affected only bFGF-loaded sponges, but not the bFGF-Heparin loaded sponges. Standard errors do not exceed 5% of values.

or subacute toxicity), the angiogenic response induced by bFGF was effectively prevented. Sponges were in fact indistinguishable from controls both at visual inspection and histological examination, neovessel formation being negligible and cellular infiltration minimal (Figure 1c). The very low Hb content of sponges after suramin treatment further confirmed the absence of angiogenesis (Figure 2a). Conversely, suramin dose of 100 mg $kg^{-1}$ given at day 1, or a dose of 200 mg $kg^{-1}$ administered at day 7, did not significantly affect neovascularisation in sponges (Table I) as evaluated histologically or via the Hb content.

bFGF has been demonstrated to interact with heparin and heparan sulphate forming a complex reported to be more resistant than bFGF alone to proteolytic degradation (Saksela *et al.*, 1988; Flaumenhaft *et al.*, 1990). Table II shows the angiogenic response elicited in sponges pre-loaded with bFGF complexed with heparin in a ratio of 1:1. Whereas heparin alone up to 10 µg/sponge had no detectable angiogenic activity, sponges loaded with the bFGF-heparin complex induced a much higher angiogenic response in comparison to bFGF alone. Angiogenesis with bFGF-heparin complex was dose dependent in the tested range of 2.5 to 10 µg/sponge. Sponges loaded with the bFGF-heparin complex showed at the dose of 10 µg of 2-fold higher Hb content compared to that observed in sponges loaded with bFGF alone, whereas in sponges loaded with heparin only no Hb was detected (Figure 2b). Results similar to those obtained with the heparin complex were obtained complexing bFGF with heparan sulphate (data not shown).

In contrast to what observed with sponges loaded with bFGF alone, suramin administered i.v. at 200 mg $kg^{-1}$ at day 1 was unable to inhibit neovascularisation induced by bFGF-heparin sponges. In fact the angiogenic score (Table II) and

**Table II** Effect of suramin on angiogenesis induced by sponges loaded with complex bFGF-Heparin

| *bFGF/sponge µg/sponge* | | *Suramin treatment mg kg⁻¹* | *day* | *Angiogenic score* |
|---|---|---|---|---|
| bFGF | 10 | – | – | + + + |
| bFGF | 10 | 200 | + 1 | 0 |
| Heparin | 10 | – | – | 0 |
| bFGF-Heparin | 10 | – | – | + + + + + |
| bFGF-Heparin | 10 | 200 | + 1 | + + + + + |
| bFGF-Heparin | 2.5 | – | – | + + + + |
| bFGF-Heparin | 2.5 | 200 | + 1 | + + + + |

Mice bearing bFGF-treated sponges were sacrificed 2 weeks after the implant. Suramin was given i.v.

the Hb content of sponges (Figure 2b) were not significantly modified in comparison to controls.

### *Tumour-induced angiogenesis*

Preliminary experiments in this laboratory had shown that conditioned medium from cultures of M5076 cells contained measurable amounts of bFGF and that it was consistently angiogenic in the rabbit cornea assay. For these reasons the M5076 tumour was chosen for *in vivo* studies to investigate the possible role of bFGF in tumour angiogenesis and to evaluate the activity of suramin on this process.

In preliminary experiments, different numbers of M5076 tumour cells (from $10^4$ to $10^6$/sponge) were entrapped in gelfoam sponges and then implanted subcutaneously and angiogenesis was evaluated at different times after implant. An inoculum of $5 \times 10^5$ cells/sponge was chosen for the subsequent experiments, since it resulted in a clear angiogenic response detectable already at 5–7 days from implant and that was maximal on 10–15 days (Figure 3a). Newly formed blood vessels of various size and length were observed within the sponge containing the entrapped tumour cells. Administration of a single dose of 200 mg $kg^{-1}$ i.v. suramin 1 day after sponge implantation significantly delayed M5076 cells induced neovascularisation (Figure 3b). In fact, after suramin treatment, an histologically detectable angiogenesis appeared only 12–15 days from implant, whereas in untreated control animals it was already evident at day 5 and maximal on day 15.

The effect of suramin treatment on the *in vivo* growth of the M5076 tumour was then investigated and Figure 4a shows the results of different treatment schedules with this drug. Single suramin doses of 200 and 150 mg $kg^{-1}$ administered i.v. 24 h after s.c. tumour transplant were able to reduce dose-dependently the growth rate of the tumour, whereas 100 mg $kg^{-1}$ had no significant effect (not shown). Suramin was active only when administered in the first days after tumour cell transplantation and in fact, no tumour growth inhibition was observed when 200 mg $kg^{-1}$ of suramin was injected on day 10, a time when the tumour was already palpable.

The growth of the M5076 tumour was enhanced by the administration of bFGF. In fact, peritumoural injections with 5 µg of bFGF given daily from day 1 after tumour transplant to day 7, significantly increased tumour growth (Figure 4b ). In addition, the growth inhibitory effect of suramin was almost completely counteracted by topical bFGF treatment. In fact, after 20–24 days from the implant, tumours in animals treated with suramin and peritumour bFGF reached the same size as untreated controls.

### Discussion

The involvement of bFGF in the angiogenetic process is well documented by studies showing bFGF stimulation of endothelial cell proliferation and motility *in vitro* (Folkman *et al.*, 1988), induction of angiogenesis in the chick embryo chorioallantoic membrane, and in the rabbit cornea assay

**Figure 3** Tumour-induced angiogenesis in sponges loaded with M5076 cells. An evident angiogenic response is visible after 15 days from implant of the sponge loaded with M5076 cells. **a**, Suramin treatment inhibit tumour-induced neovascularisation **b**.

(Folkman & Klagsbrun, 1987). Mice bearing s.c. gelfoam sponges loaded with bFGF developed a clear angiogenic response within the implant. This response was dramatically reduced by the systemic administration of suramin as determined by histological and biochemical parameters. In this model suramin exerted maximal activity if administered on the first day after implant, whereas no effect was seen if the compound was given on day 7 at the dose fully inhibitory on day 1. Taking also in account that suramin is endowed with an extremely long half-life in the body (Stein *et al.*, 1989), these findings indicate that in this experimental conditions suramin interferes preferentially with the initial phases of the angiogenetic process, whereas it lost effectiveness when the process was already established. Previous studies (Coffey *et al.*, 1987) have shown that suramin by binding bFGF prevented the interaction of this growth factor with its receptors. Moreover, in our hands suramin was not cytotoxic *in vitro* for resting or proliferating endothelial cells even after long (96 h) exposure times. These observations suggest that also in the *in vivo* experimental model employed endothelial cells were not directly affected in their viability by suramin. The finding that suramin was only active in the early phase of angiogenesis is a further, albeit indirect, support for the contention that in the bFGF-sponge model, suramin activity derives from its capacity to bind and inactivate bFGF, a key factors in stimulating endothelial cells multiplication and thus in angiogenesis.

In physiological conditions bFGF present in tissues is essentially bound to heparan sulphates associated to the extracellular matrix (Folkman *et al.*, 1988), and is released complexed to heparan sulphates after degradation of the extracellular matrix (Saksela *et al.*, 1988). This complex has been shown to be more resistant to protease degradation and to better diffuse in the extracellular environment than free bFGF (Flaumenhaft *et al.*, 1990). In line with the above observations, the angiogenetic response elicited by bFGF when complexed to heparin or heparan sulphate was much higher than seen in sponges with free bFGF. Suramin was inactive against the bFGF-heparin complex suggesting that heparin might compete for the binding to bFGF, with a much higher affinity than suramin. Importantly however, while the biological functions of bFGF are maintained and enhanced after binding to heparin, the binding with suramin impairs bFGF activity.

**Figure 4** Effect of suramin on the growth of s.c. M5076 tumour. Suramin had maximal activity at the dose of 200 mg kg$^{-1}$ i.v. Treatment performed at day 10 from tumour implant had no activity. The differences between suramin 150–200 mg kg$^{-1}$ and controls are significant as $P < 0.01$ (Student $t$ test) **a**. Exogenous administration of bFGF (5 μg for 7 days) enhanced tumour growth and impaired Suramin effect. Control animals were treated with PBS + BSA 0.1% **b**. Standard errors do not exceed 5% of values.

Since it has been advanced that bFGF plays a key role also in tumour induced neovascularisation (Folkman *et al.*, 1988; Klagsbrun *et al.*, 1986), and, as recently described (Hori *et al.*, 1991; Gross *et al.*, 1990), in tumour growth, it was of interest to investigate suramin in a simplified model of tumour-associated angiogenesis as that represented by M5076 cells entrapped in gelfoam sponges. Results obtained were similar to those seen with bFGF sponges since suramin was effective only when given within 24–48 h after implantation, i.e. during the early stages of the response.

Suramin was also able to reduce the growth of M5076 tumour transplanted subcutaneously, with the maximal activity seen administering the compound on the first day after tumour transplantation. In this sytem, repeated peritumoural injections of bFGF increased the tumour growth rate, and significantly reduced the tumour-growth inhibitory activity of suramin. Since suramin up to the concentration of 150 μg ml$^{-1}$ (data not shown) was not cytotoxic to cultured M5076 cells, the hypothesis can be advanced that suramin delays M5076 tumour growth not via a direct cytotoxicity on neoplastic cells, but indirectly through interference with neovessel formation elicited by tumour-produced bFGF. It should be noted however that the role of other growth factors involved in angiogenesis (e.g. PDGF, TGFβ, etc.), was not evaluated in this study; accordingly it cannot be excluded that suramin also interferes with their activity in tumour growth.

In conclusion, these results provide further direct support to the conclusion that a major component in the antineoplastic activity of suramin may be an interference with neovascularisation induced by growth factors such as bFGF, produced by the neoplastic cells. Since suramin administration is associated with important toxicities in both animals and humans (Stein *et al.*, 1989; La Rocca *et al.*, 1990), the identification of novel molecules capable of interfering with tumour-induced angiogenesis, but possessing a more favourable therapeutic index, could open alternative approaches to the treatment of solid neoplasms.

### References

AUSPRUNK, D.H. & FOLKMAN, J. (1977). Migration and proliferation of endothelial cells in preformed and newly formed blood vessels during tumour angiogenesis. *Microvasc. Res.*, **14**, 53–65.

BREM, S.S., GULLINO, P.M. & MEDINA, D. (1977). Angiogenesis: a marker for neoplastic transformation of mammary capillary hyperplasia. *Science*, **195**, 880–882.

COFFEY, R.J., LEOF, E.B., SHIPLEY, G.D. & MOSES, M.L. (1987). Suramin inhibition of growth factor receptor binding and mitogenicitiy in ARK-2B cells. *J. Cell Physiol.*, **132**, 143–148.

FLAUMENHAFT, R., MOSCATELLI, D. & RIFKIN, D.B. (1990). Heparin and heparan sulfate increase the radius of diffusion and action of basic fibroblast growth factor. *J. Cell Biol.*, **111**, 1615–1659.

FOLKMAN, J. (1990). What is the evidence that tumours are angiogenesis dependent? *J. Natl Cancer Inst.*, **82**, 4–6.

FOLKMAN, J. & KLAGSBRUN, M. (1987). Angiogenic factors. *Science*, **235**, 442–447.

FOLKMAN, J., KLAGSBRUN, M., SASSE, J., WADZINSKI, M., INGBER, D. & VLODAVSKY, I. (1988). A heparin-binding angiogenic protein – basic fibroblast growth factor – is stored within basement membrane. *Am. J. Pathol.*, **130**, 393–400.

FOLKMAN, J.K, WATSON, K., INGBER, D. & HANAHAN, D. (1989). Induction of angiogenesis during the transition from hyperplasia to neoplasia. *Nature*, **339**, 58–61.

GERAN, R.I., GREENBERG, N.H., MCDONALD, M.M., SCHUMAKER, A.M. & ABBOTT, B.J. (1972). Protocols for screening chemical agents and natural products against animal tumours and other biological systems. *Cancer Chem. Rep.*, Part 3, 1–103.

GOLDIE, I. (1969). The synovial microvascular derangement in rheumatoid arthritis and osteo-arthritis. *Acta Orthop. Scan.*, **40**, 751–766.

GOSPODAROWICZ, D. & THAKRAL, K.K. (1978). Production of a corpus luteum angiogenic factor responsible for proliferation of capillaries and neovascularization of the corpus luteum. *Proc. Natl Acad. Sci. USA*, **75**, 847–851.

GROSS, J.L., HERBLIN, W.F., DUSAK, B.A., CZERNIAK, P., DIARENS, M. & DEXTER, D.L. (1990). Modulation of solid tumor growth *in vivo* by bFGF. *Proc. Am. Ass. Cancer Res.*, **31**, 79.

GULLINO, P.M. (1978). Angiogenesis and oncogenesis. *J. Natl Cancer Inst.*, **61**, 639.

HAWKING, F. (1987). Suramin with special reference to onchocerciasis. *Adv. Pharmacol. Chemother.*, **15**, 289–322.

HAYEK, A., CULLER, F.L., BEATTIE, G.M., LOPEZ, A.D., LUEVAS, P. & BAIRD, A. (1987). An *in vivo* model for study the angiogenic effects of basic fibroblast growth factor. *Biochem. Biophys. Res. Commun.*, **147**, 876–880.

HORI, A., SASADA, R., MATSUTANI, E., NAITO, K., SAKURA, Y., FUJITA, T. & KOZAI, Y. (1991). Suppression of solid tumor growth by immunoneutralizing monoclonal antibody against human basic fibroblast growth factor. *Cancer Res.*, **51**, 6180.

HOSANG, M. (1985). Suramin binds to platelet-derived growth factor and inhibits its biological activity. *J. Cell. Biochem.*, **29**, 265–273.

KLAGSBRUN, M., SASSE, J., SULLIVAN, R. & SMITH, J.A. (1986). Human tumour cells synthesize an endothelial cell growth factor that is structurally related to basic fibroblast growth factor. *Proc. Natl Acad. Sci. USA*, **83**, 2448–2352.

KIM, J.H., SHERWOOD, E.R., SUTKOWSKI, D.M., LEE, C. & KOZLOWSKI, J.M. (1991). Inhibition of prostatic tumour cells proliferation by suramin: alterations in TGF alpha-mediated autocrine growth regulation and cell cycle distribution. *J. Urol.*, **146**, 171–176.

KNIGHTON, D.R., SILVER, I.A. & FOLKMAN, J. (1981). Regulation of wound healing angiogenesis. Effect of oxygen gradients in inspired oxygen concentration. *Surgery*, **90**, 262–270.

LA ROCCA, R.V., MEER, J. & GILLIAT, D.M. (1990). Suramin-induced polyneuropathy. *Neurol.*, **40**, 954–960.

LA ROCCA, R.V., MEYERS, C.E., STEIN, C.A., COOPER, M.R. & UHRICH, M. (1990). Effect of suramin in patients with refractory nodular lymphomas requiring systemic therapy. *Proc. Am. Soc. Clin. Oncol.*, **9**, 1041.

MILLS, G.B., ZHANG, N., MAY, C., HILL, M. & CHUNG, A. (1990). Suramin prevents binding of interleukin 2 to its cell surface receptor: a possible mechanism for immunosuppression. *Cancer Res.*, **50**, 3036–3042.

MYERS, C.E., LA ROCCA, R.V., STEIN, C.A., COOPER, M.R.,, DAWSON, N., CHOYKE, P., LINEHAN, M. & UHRICH, M. (1990). Treatment of hormonally refractory prostate cancer with suramin. *Proc. Am. Soc. Clin. Oncol.*, **9**, 133.

PIENTA, K.J., ISAACS, W.B., VINDIVICH, D. & COFFEY, D.S. (1991). The effects of basic fibroblast growth factor and suramin on cell motility and growth of rat prostate cancer cells. *J. Urol.*, **145**, 199–202.

POLLACK, M. & RICHARD, M. (1990). Suramin blockade of insulin-like growth factor 1-stimulated proliferation of human osteosarcoma cells. *J. Natl Cancer Inst.*, **82**, 1349–1352.

RIFKIN, D.B. & MOSCATELLI, D. (1989). Recent developments in the cell biology of basic fibroblast growth factor. *J. Cell. Biol.*, **109**, 1–6.

SAKSELA, O., MOSCATELLI, D., SOMMER, A. & RIFKIN, D.B. (1988). Endothelial cell-derived heparan sulfate binds basic fibroblast growth factor and protects it from proteolytic degradation. *J. Cell Biol.*, **107**, 743–751.

SHING, Y., FOLKMAN, J., HAUDENSCHILD, C., LUND, D., CRUM, R. & KLAGSBRUN, M. (1985). Angiogenesis is stimulated by a tumour-derived endothelial cell growth factor. *J. Cell Biochem.*, **29**, 275–287.

SPIGELMAN, Z., DOWERS, A., KENNEDY, S., DI SORBO, D., O'BRIEN, M., BAR, R. & MCCAFFREY, R. (1987). Antiproliferative effects of suramin on lymphoid cells. *Cancer Res.*, **47**, 4694–4698.

STEIN, C.A., LA ROCCA, R.V., THOMAS, R., MCATEE, N. & MYERS, L.E. (1989). Suramin: an anticancer drug with a unique mechanism of action. *J. Clin. Oncol.*, **7**, 499–508.

TALMADGE, J.E., KEY, M.E. & HART, I.R. (1981). Characterization of a murine ovarian reticulum cell sarcoma of histiocytic origin. *Cancer Res.*, **41**, 1271–1280.

THOMPSON, J.A., ANDERSON, K.D., DIPIETRO, J.M., ZWIEBEL, J.A., ZAMETTA, M., FRENCH ANDERSON, W. & MACIAG, T. (1988). Site-directed neovessel formation *in vivo*. *Science*, **241**, 1349–1352.